# Probing the Informational and Regulatory Plasticity of a Transcription Factor DNA–Binding Domain

**DOI:** 10.1371/journal.pgen.1002614

**Published:** 2012-03-29

**Authors:** Ryan K. Shultzaberger, Sebastian J. Maerkl, Jack F. Kirsch, Michael B. Eisen

**Affiliations:** 1 Department of Molecular and Cell Biology, University of California Berkeley, Berkeley, California, United States of America; 2 School of Engineering, Institute of Bioengineering, Ecole Polytechnique Federale de Lausanne (EPFL), Lausanne, Switzerland; 3 Department of Genome Sciences, Genomic Division, Ernest Orlando Lawrence Berkeley National Lab, Berkeley, California, United States of America; 4 Howard Hughes Medical Institute, University of California Berkeley, Berkeley, California, United States of America; University of California San Francisco, United States of America

## Abstract

Transcription factors have two functional constraints on their evolution: (1) their binding sites must have enough information to be distinguishable from all other sequences in the genome, and (2) they must bind these sites with an affinity that appropriately modulates the rate of transcription. Since both are determined by the biophysical properties of the DNA–binding domain, selection on one will ultimately affect the other. We were interested in understanding how plastic the informational and regulatory properties of a transcription factor are and how transcription factors evolve to balance these constraints. To study this, we developed an *in vivo* selection system in *Escherichia coli* to identify variants of the helix-turn-helix transcription factor MarA that bind different sets of binding sites with varying degrees of degeneracy. Unlike previous *in vitro* methods used to identify novel DNA binders and to probe the plasticity of the binding domain, our selections were done within the context of the initiation complex, selecting for both specific binding within the genome and for a physiologically significant strength of interaction to maintain function of the factor. Using MITOMI, quantitative PCR, and a binding site fitness assay, we characterized the binding, function, and fitness of some of these variants. We observed that a large range of binding preferences, information contents, and activities could be accessed with a few mutations, suggesting that transcriptional regulatory networks are highly adaptable and expandable.

## Author Summary

The main role of transcription factors is to modulate the expression levels of functionally related genes in response to environmental and cellular cues. For this process to be precise, the transcription factor needs to locate and bind specific DNA sequences in the genome and needs to bind these sites with a strength that appropriately adjusts the amount of gene expressed. Both specific protein–DNA interactions and transcription factor activity are intimately coupled, because they are both dependent upon the biochemical properties of the DNA–binding domain. Here we experimentally probe how variable these properties are using a novel *in vivo* selection assay. We observed that the specific binding preferences for the transcription factor MarA and its transcriptional activity can be altered over a large range with a few mutations and that selection on one function will impact the other. This work helps us to better understand the mechanism of transcriptional regulation and its evolution, and may prove useful for the engineering of transcription factors and regulatory networks.

## Introduction

The precise regulation of gene expression depends upon the specific binding of transcription factors to their cognate binding sites. For this process to be accurate, the sites for each factor need to be separable from all other sequences in the genome [1], [2]. Many groups have studied specific protein-DNA interactions, and while nucleotide preferences are starting to be understood at the biophysical level for some DNA binding domains [3]–[5], no universal DNA-recognition code has been discovered [6]. What has emerged is a consistent picture of binding site degeneracy. That is, for most factors there is a single consensus binding site that is bound with the highest affinity and an increasing number of lower affinity sites that vary from the consensus. At some point the degeneration is so great that all remaining sites show the same non-specific binding energy [7]–[9]. Using information theory, the amount of conservation within a set of binding sites (information content), as well as the amount of information needed to specifically locate *N* sites in a genome of length *L*, can be quantified [1], [10]. In bacteria, it has been shown that these values are identical for many factors, suggesting that the size of a factor's regulon constrains how specific it needs to be [1], [11], [12]. This relationship does not hold as well for individual transcription factors in eukaryotes though [13], [14], where gene regulation is often under the control of cooperatively acting factors [15].

Once bound to their target sequence, transcription factors can modulate the rate of expression over a range of activities. Differences in expression levels have been suggested and shown to vary with binding site strength [16]–[19]. Given this relationship, the range and continuity of binding affinities for a factor partially define the range and continuity of potential outputs for that factor [19], [20]. These outputs in turn can significantly affect the phenotype and fitness of the cell and are selected to maximize cellular gain while minimizing cost [19], [21], [22]. Therefore, there is not only a selective advantage for transcription factors to specifically recognize and bind their target sites, but to bind them with an affinity that produces the maximally fit transcriptional output. Since both specific binding preferences and transcriptional activity are dependent on the distribution of binding energies for a factor, selection on one will ultimately affect the other.

We are interested in understanding how plastic the informational and regulatory properties of a transcription factor are, and how transcription factors evolve to balance these functions. To address this, we developed an *in vivo* selection system in *E. coli* to select for functional variants of the transcription factor MarA with altered binding preferences, whose binding properties and activity could be further characterized. By *functional*, we mean that a variant could modulate the level of transcriptional output within a physiological range. This is in contrast to *in vitro* selection assays, like phage display, that generally select for high affinity binding to a single target sequence, and disregard the impact of these mutations on transcriptional activity.

To do these selections, we wanted to use a monomeric, transcriptional activator whose binding sites have been characterized and structure had been solved. MarA fit these criteria. It is a monomeric, helix-turn-helix transcription factor in the AraC family [23] that can both activate and repress transcription in *E. coli* [24]–[26]. It regulates the expression of approximately 20 genes involved in exporting low levels of drugs and organic solvents from the cell [24], [27]. The structure of the MarA-DNA complex suggests that specific recognition occurs through two alpha-helices that bind the major groove [28], [29]. Additionally, MarA has two homologues in *E. coli*, Rob and SoxS, that have similar binding preferences [30], suggesting that the MarA binding domain can be selected to recognize additional sites.

## Results

### MarA binding domain and sites

**Figure 1:**
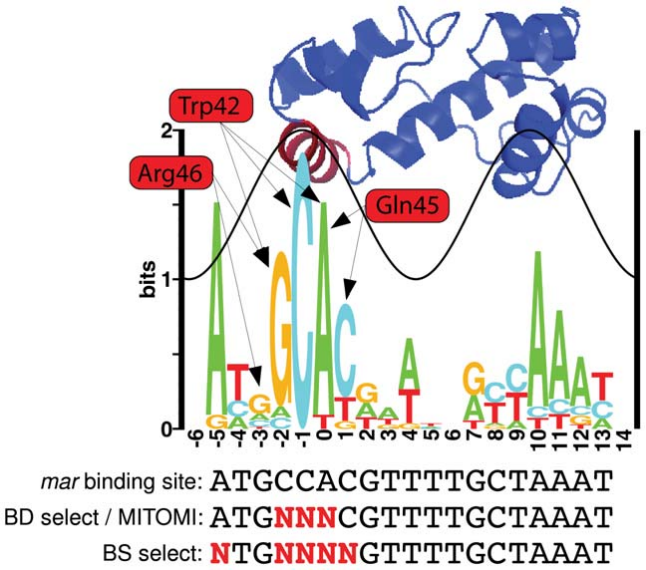
MarA logo and structure. The height of each letter in the sequence logo is proportional to the frequency of that base at that position. The height of the stack at each position is the information content [39]. The sine wave on the logo has the same helical twist as B-form DNA (10.6 bp) [32] and its position was assigned based on the MarA-DNA cocrystal structure [28]. The structure of *E. coli* MarA is positioned above the logo to show which bases each helix specifically binds [28]. Three residues in helix 3 (red helix on the structure) specifically contact DNA bases. Arrows show which bases these residues specify. We randomized these three residues and selected for variants that had altered affinity. Binding domain selections (BD select), MITOMI experiments, and *in vivo* binding site selections (BS select) were performed with variants of the *mar* MarA binding site. A red ‘N’ specifies bases that were varied for each experiment. For binding site and binding domain selections, these variants were cloned into the selection plasmid in the MarA binding site (Figure 2B). The information content for this logo is 12.6
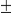
0.9 bits.

We generated a sequence logo from the 16 *E. coli* MarA binding sites summarized in Martin *et al.* [24] to visualize the natural binding preference of the protein and the relative contribution of each contacting residue to binding specificity (Figure 1). Sequence conservation follows a sine wave as seen for other transcription factors [31], [32]. MarA specifically contacts the DNA through helices 3 and 6. Bases contacted by helix 3 (red helix on structure, DNA positions 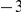 to 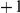) have a greater information content than do those contacted by helix 6 (blue helix on structure that intersects the sine wave, DNA positions 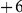 to 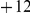), suggesting that helix 3 is more important for specific DNA recognition. This is consistent with alanine-scanning mutagenesis data for MarA [33].

Three residues in helix 3 (Trp42, Gln45, and Arg46) specifically contact DNA bases according to the MarA-DNA structure [28] (Figure 1). Interestingly, the structure does not predict a specific contact at position 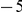, but the sequence logo indicates a strong preference for ‘A’ at this position. The ‘C’ at position 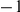 is completely conserved and only contacted by the tryptophan at residue 42, suggesting this is a highly specific amino acid.

### Selection of MarA binding domain variants

**Figure 2:**
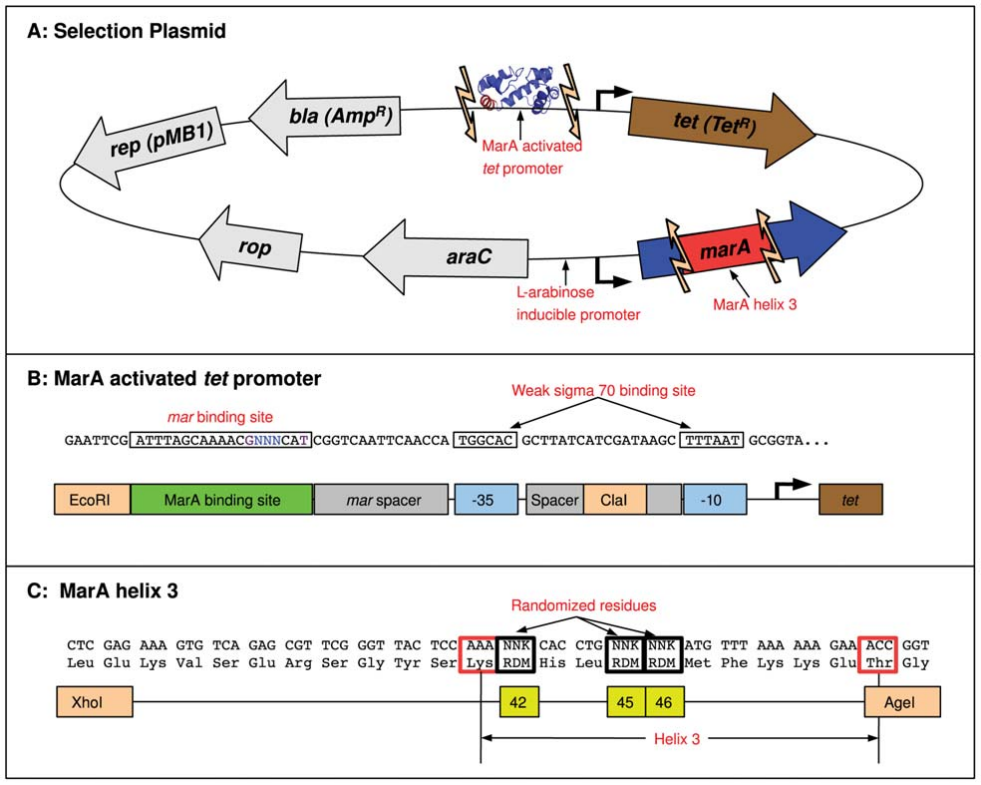
MarA binding domain selection system. (A) A schematic representation of the MarA selection plasmid. Expression of the *marA* gene is controlled by an AraC repressed, L-arabinose inducible promoter [34]. Unique restriction sites (light orange arrows) flank the promoter region of the tetracycline resistance gene *tet* and helix 3 of the *marA* gene. Promoter and binding domain variants can be cloned into this plasmid and functional binding domain-binding site pairs can be identified by selection in tetracycline+L-arabinose. (B) The sequence of the MarA-activated *tet* promoter (top) and a cartoon marking each component (bottom). This construct is based on the promoter used in [19]. Bases that were varied in binding domain selection experiments are designated by a blue ‘N’. Additional bases that were randomized in the binding site selection are shown in purple. Orange boxes mark the restriction sites used to clone in these constructs. The *mar* binding site has the opposite orientation as in Figure 1. (C) The sequence of the MarA binding domain variants (top) and a cartoon marking components within this region. The three residues that were randomized are marked with yellow boxes. The boundaries of helix 3 are marked with red boxes.

To identify variants of MarA that have altered binding preferences, we randomized the three specifically contacting residues in helix 3 and selected for mutants that could bind a target DNA sequence and initiate transcription of the tetracycline resistance gene (*tet*) on the selection plasmid shown in Figure 2. Both the promoter of the *tet* gene and helix 3 of the MarA protein were flanked by restriction sites that allowed promoter and binding domain variants to be cloned into the plasmid (Figure 2). Functional MarA protein-binding site pairs within this system activated *tet* and allowed for cell survival in tetracycline. As we increased the concentration of drug, we selected for higher affinity interactions [19]. Additional parameters can affect the rate of transcriptional initiation, most notably the position of the binding site relative to the polymerase [24]. Since we vary the binding site within a fixed promoter context, our selection should just be on the strength of the DNA-protein interaction. We performed our selection in the *E. coli* strain N8453 (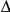 *mar*, 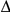 *sox-8*::*cat*, 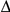 *rob*::*kan*, see Materials and Methods) to prevent activation by wild type MarA, or by the MarA *E. coli* homologues Rob and SoxS. Expression of MarA on the plasmid was controlled by an L-arabinose inducible promoter [34].

We needed to identify a 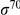 promoter that was only functional when activated to have *tet* expression and cell survival dependent upon MarA binding. To identify one, we randomized the 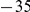 of the *tet* promoter construct (Figure 2B) and selected for a promoter sequence that allowed cell growth on tetracycline plates with L-arabinose (induced expression of MarA) but not on plates without it (see Materials and Methods). The 6.5 bit 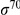 binding site that we identified is marked in Figure 2B. The strength of this site was predicted using the model presented in [18], and is an average site compared to all 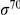 sites in the genome. In a single construct, we cloned 3 in-frame and 2 out-of-frame stop codons into helix 3 of the MarA binding domain and tested if the resulting truncated protein could express *tet* with this promoter. At 15 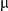
g/ml tetracycline and 0.1% L-arabinose, we observed significant growth with wild type MarA, and no growth with the truncated mutant (data not shown), suggesting that in this condition activation of *tet* and cell survival is dependent upon binding by MarA.

The MarA regulon in *E. coli* includes the *arcAB* operon, which when over-expressed shows increased tolerance to many antibiotics including tetracylcine [35], [36]. To ensure that we are selecting for variants that directly activate *tet*, we performed a selection against the anti-consensus MarA binding site (the worst possible binding site according to Figure 1: CGTTTGACCCGCCAGGGCG). We could not identify any protein variants that allowed for survival in 20 or 30 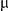
g/ml tetracycline, suggesting that differential regulation of the MarA regulon is not sufficient for cell viability. This does not exclude the possibility that the over-expression of the *arcAB* operon may reduce the selective pressure on *tet* production. Selection in this system is somewhat similar to selection in a natural system, where the fitness of a binder is dependent upon the relative contribution of multiply expressed genes. We have in essence added *tet* to the MarA regulon. Because of the high concentration of tetracycline used for selection, the fitness gain for expressing *tet* is probably much greater than for any other gene that it regulates.

MarA binding domain mutants were selected against three variants of the 15.3 bit *mar* binding site (Figure 1) that is found upstream of the *mar* operon in *E. coli* [24]. The three target sequences we selected against are named ‘GCA’, ‘GAA’ and ‘GAC’ according to the bases present at positions 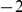, 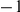 and 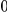 (Figure 1 and Figure 2B). We varied these bases because they are the most highly conserved ones contacted by helix 3. Binding domain libraries were made as described in Materials and Methods. We transformed the N8453 cells with each library and selected for growth on plates at 20 and 30 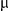
g/ml of tetracycline +0.1% L-arabinose. Individual colonies were sequenced.

**Figure 3:**
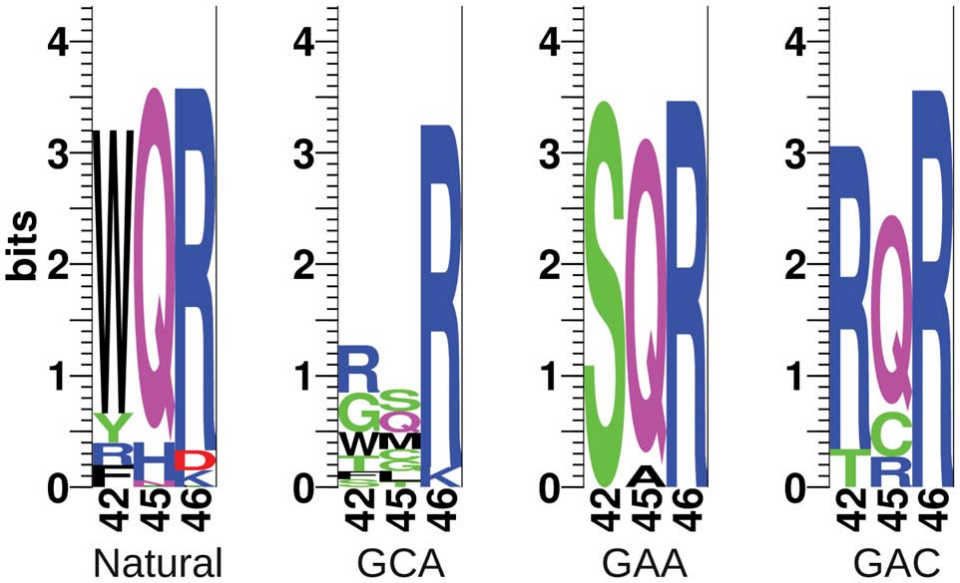
Sequence logos of natural and selected variants of the MarA binding domain. The sequence logos show the degree of variability at residues 42, 45 and 46 in functional binders selected against different binding sites. ‘Natural’ is the natural variability in positions 42, 45 and 46 for MarA homologues. ‘GCA’, ‘GAA’ and ‘GAC’ designate which sequence the binding domain was selected against. These logos are made from the sequences in Table 1.

Sequences of viable MarA binding domain variants are shown in Table 1 and sequence logos generated from these variants are shown in Figure 3. Each binding domain is referenced by residues 42, 45 and 46. For example, wild type MarA is noted as WQR. Of the 18 sequenced binding domains selected against the MarA consensus ‘GCA’ binding site at 20 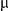
g/ml tetracycline, we identified 13 different variants, including that of the wild type protein, that could initiate *tet* transcription to a sufficiently high level for cell survival. Only 5 different variants were observed at 30 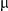
g/ml tetracycline, and no new variants were observed at this higher concentration as expected. Three of the 13 binding domains were represented by multiple codon sets further supporting that these variants are functional. Interestingly, only the ‘TCK’ variant selected against ‘GCA’ lacks an arginine at position 46, but it retains a positively charged lysine residue at that position.

**Table 1:** Functional MarA binding domain variants selected against different binding sites.

BS	42-45-46	Tet-20	Tet-30	Total	Codons	Best	MITOMI
GCA	R-Q-R	2	3	5	1	X	X
GCA	R-G-R	1	3	4	1		
GCA	R-L-R	2	1	3	2		
GCA	R-T-R	1	0	1	1		X
GCA	G-S-R	3	1	4	2		
GCA	G-Q-R	1	1	2	2		
GCA	G-G-R	1	0	1	1		
**GCA**	**W-Q-R**	**1**	**0**	**1**	**1**		**X**
GCA	W-M-R	2	0	2	1		
GCA	T-S-R	1	0	1	1		
GCA	T-C-K	1	0	1	1		
GCA	S-C-R	1	0	1	1		
GCA	F-M-R	1	0	1	1		
GAA	S-A-R	1	0	1	1		X
GAA	S-Q-R	15	15	30	3	X	
GAC	R-C-R	3	0	3	1		
GAC	R-Q-R	13	0	12	1		
GAC	T-R-R	2	0	2	1	X	X

**Figure 4:**
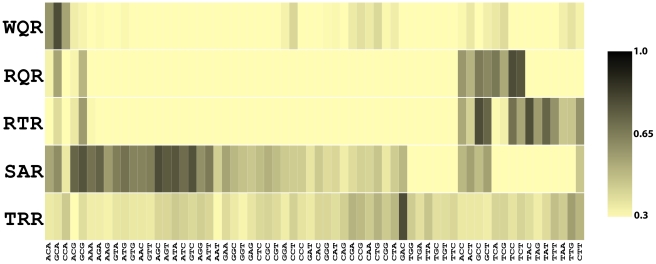
Binding affinities for 5 MarA variants to 64 binding sites. The heat map shows the relative binding affinities of wild type MarA (WQR) and 4 selected variants to 64 variations of the *mar* binding site (Figure 1). Each MarA variant (y-axis) is named according to its residues at positions 42, 45 and 46. Each DNA sequence (x-axis) was substituted for ‘NNN’ in the *mar* binding site (Figure 1). Data for all variants were normalized so the highest affinity site was set to 1 (black). All other sites are colored relative to that site according to the color scale. All sites below 0.3 were colored the same as 0.3.

Each row represents a different MarA protein variant that will initiate transcription in our selection system. ‘BS’ is the binding site the MarA variant was selected against. The three letters correspond to the bases at position 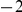, 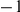 and 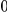 in the *mar* binding site (Figure 1, Figure 2B). ‘42-45-46’ are the residues at positions 42, 45 and 46 in the selected MarA variants (Figure 2C). ‘Tet-20’ and ‘Tet-30’ are the number of colonies selected at that tetracycline concentration that contained that variant. ‘Total’ is the sum of ‘Tet-20’ and ‘Tet-30’. ‘Codons’ are the number of different codons sets that specified that variant. An ‘X’ in the ‘Best’ column identifies the variant that had the highest affinity for a given binding site as determined by a competition experiment. An ‘X’ in the ‘MITOMI’ column identifies the protein variants whose binding we characterized by MITOMI (Figure 4) and by an *in vivo* binding site selection (Figure 7). The variant that corresponds to the wild type protein (WQR) is bolded.

Selection against the ‘GAA’ and ‘GAC’ binding sites showed much less variability in the number of identified functional MarA variants. We only identified two mutants that could activate the ‘GAA’ binding site and three that could activate ‘GAC’. No colonies were observed when we selected against ‘GAC’ at the higher tetracycline concentration of 30 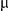
g/ml.

We were interested in how the variability in the selected mutants compared to the natural variability at these residues. We blasted the *E. coli* MarA sequence against all bacterial genomes using BlastP with non-redundant protein sequences and default search parameters [37]. The top 250 hits were aligned by ClustalX [38] and sequence logos were generated using the Delila programs [39] (Figure 3, Natural). Both the natural and the experimentally selected binding domain variants show a strong preference for arginine at position 46. Interestingly, tryptophan is highly conserved at position 42 in the natural binding domains, whereas it was only observed in two selected variants (Table 1). In a similar selection for specifically contacting residues in the engrailed homeodomain by phage display, experimentally and naturally selected variability correlated well [40]. Engrailed binds a more specific set of sequences than does MarA. Therefore, natural selection on binding by engrailed is probably directed to maintain high affinity to a single or small set of sites as was experimentally selected. Conversely, MarA has probably been selected to maintain affinity to a more degenerate set of sequences, which may explain the discordance between the naturally and experimentally selected binding domains.

To identify the highest affinity MarA mutant for each of the three DNA binding sites, the protein binding domains in each library were competed against each other in liquid culture containing 30 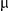
g/ml tetracycline+L-arabinose for 24 hours. The competed cultures were mini-prepped, retransformed and individual variants were sequenced (Materials and Methods). We expected the mutant that produced the highest *tet* output to be represented at the highest frequency in the competed population as seen in a similar experiment [19]. We sequenced 8 individuals from each library and observed only one protein variant for each target binding site: RQR for ‘GCA’, SQR for ‘GAA’ and TRR for ‘GAC’ (Table 1, marked with ‘X’ in Best column). Interestingly, wild type MarA (WQR) was not identified as the most fit variant for its naturally evolved consensus binding site ‘GCA’.

### High-throughput measurement of DNA binding preferences for MarA mutants

We determined the relative affinity of wild type MarA and four selected MarA variants to 64 different binding sites using MITOMI (Figure 4). MITOMI (Mechanically Induced Trapping of Molecular Interactions) measures the relative thermodynamic association constant of a single transcription factor for a large number of DNA sequences using a microfluidics based approach. The relative amount of fluorescently-labeled protein associated with fluorescently-labeled DNA is quantified by microscopy for each binding site to determine interaction strengths [8].

**Figure 5:**
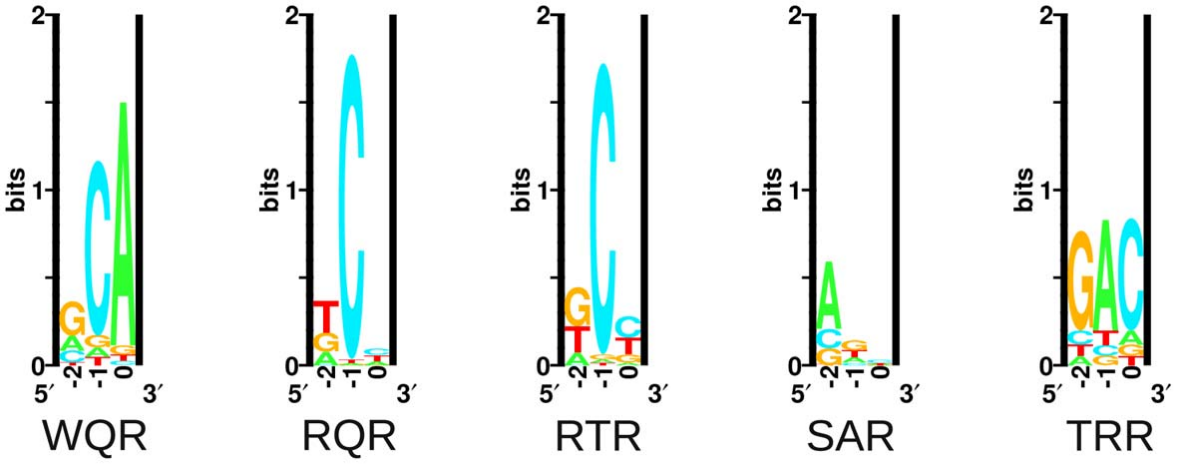
Energy logos for MarA variant binding sites. Energy logos were generated from the MITOMI data for the 5 variants in Figure 4, using the enoLogos Webserver [42] (see Materials and Methods).

The 64 sequences we measured binding to covered all combinations of bases at positions 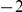, 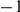 and 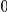 in the *mar* binding site (Figure 1). The 5 transcription factor variants chosen were wild type MarA (WQR), the most fit binder for the wild type consensus binding site (RQR), a double mutant that binds to the wild type consensus (RTR), a double mutant that activates the ‘GAA’ site (SAR), and the most fit mutant for the binding site ‘GAC’ (TRR). We did not obtain reliable binding data for SQR, the most fit mutant for ‘GAA’, and therefore did not include it in this study. For each of these five transcription factor variants, we set the binding affinity of the strongest site to 1 and scaled the strength of all other sites relative to that (Figure S1). To identify sequences that are similarly bound for each mutant, we clustered the DNA binding sites according to their relative affinities using Cluster [41] (Figure 4). Additionally, we we generated energy-based position weight matrices and logos [42] (Figure 5), and calculated the degree of similarity between all matrices as Kullback-Leibler Divergences (KLD) using the program MatCompare [43] (see Materials and Methods). A KLD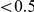 generally indicates that two matrices are significantly similar, and a KLD of 0 indicates that they are identical. All measured binding affinities, position weight matrices, and pair-wise KLD values are reported in Table S1.

MITOMI data for wild type MarA are consistent with the MarA sequence logo (Figure 1). Three sequences are tightly bound, ‘GCA’
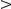
‘ACA’
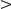
‘CCA’, as seen in natural sites. A single mutation from a Trp at position 42 to an Arg has a dramatic effect on the binding preferences of the factor (Figure 5, KLD = 1.53). The RQR mutant still specifically recognizes ‘GCA’, but with a 1.6 fold reduced affinity relative to its most tightly bound site ‘TCC’. As with wild type MarA, RQR has a strong preference for ‘C’ at position 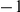, but overall RQR is a less specific binder; the information content (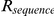) [1] for positions 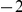 to 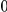 is 3.03 and 2.27 bits for WQR and RQR respectively (Figure 5, Table 2). The 2.46 bit RTR logo is significantly similar to the RQR logo (KLD = 0.15), but shows a slight decrease in degeneracy at position 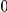, as well as a switch in preference for ‘G’ over ‘T’ at position 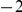. Interestingly, the RQR and RTR mutants maintained the same relative difference in affinity between the bound sequences ‘GCA’, ‘ACA’ and ‘CCA’ as wild type (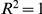 for both, data not shown), suggesting that the core binding preferences of wild type are somehow preserved in these variants although they are no longer the highest affinity sites.

**Table 2:** Specific binding energies and information contents.

Variant	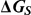	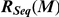	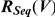	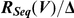 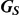
WQR	2.70	3.03	3.26	1.21
RQR	4.46	2.27	2.17	0.49
RTR	3.30	2.46	2.52	0.76
SAR*	−9.98	0.80	4.66	−0.47
TRR	2.40	2.44	6.00	2.5

**Figure 6:**
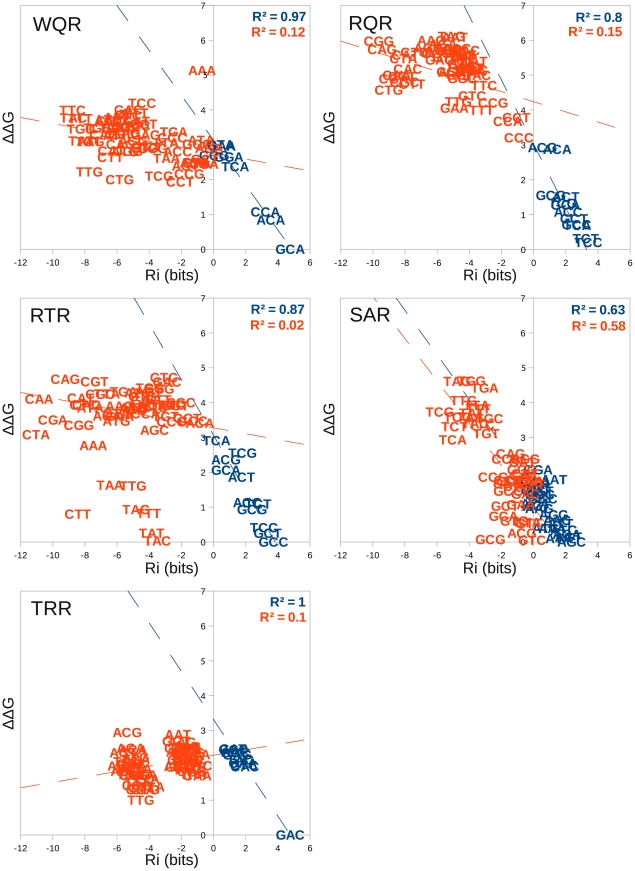
An independent and additive thermodynamic binding model fits the MITOMI data with varying degrees of success. The relative binding affinity of each mutant to each binding site (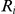) was calculated using the models presented in Figure 5. This was plotted against the 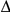 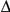 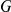 of binding for each sequence as determined by the difference in binding energy between that sequence and the highest affinity site (kJ/mol). A linear regression line was fit to sites with an 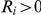 bits (blue sequences) and sites 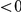 bits (red sequences). The intercept of these lines were used to approximate the boundary between specifically and non-specifically bound sites and the corresponding 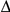 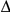 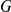 values are reported in Table 2. 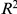 values for each regression line are given in the upper right hand corner in the same color.

**Figure 7:**
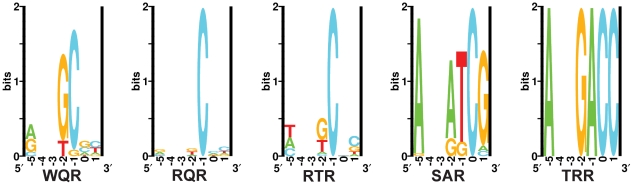
*In vivo* binding site selection logos. Sequence logos were generated from the chromatograms in Figure S2. Positions 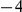 and 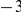 were not randomized in the selection and therefore are left blank in the logos.

‘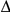 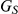
’ is the specific binding energy of the highest affinity site as determined by the intercept of the regression lines in Figure 6. ‘
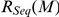
’ is the information content in bits of the corresponding energy logo for each mutant over the range of 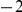 to 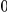 (Figure 5). ‘
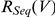
’ is the information content of the *in vivo* binding selection logo over the range of 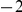 to 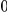 (Figure 7). * denotes that we are not confident in the 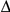 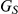 calculation for that mutant.

SAR is the least specific of the variants (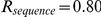 bits). It shows a preference for ‘A’ or ‘G’ at position 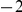, and almost no preference at positions 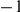 and 0. It does not strongly bind ‘GAA’, the site it was selected against. Conversely, TRR appears to only bind its selected target site ‘GAC’ (Figure S1). While TRR is specific for this sequence, the relative difference in binding strength between ‘GAC’ and the non-specific background (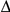 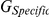) is much less than observed for WQR, RQR and RTR (Figure S1). As the logos in Figure 5 are generated from the calculated differences in binding energy from the strongest bound site to all single base-pair mutants (see Materials and Methods), a low 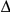 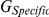 would result in a logo with a weak equiprobable conservation of all non-specifically bound bases at each position as observed for TRR.

Given the MITOMI data, we can test two assumptions that underlie most thermodynamic DNA binding models: (1) that the energetic contribution of each nucleotide at each position is independent of neighboring bases and (2) that this contribution is purely additive to the overall binding affinity [7], [44], [45]. Using **Scan**, an information theory based program that predicts binding affinities based on an independent and additive model, we calculated the predicted affinity for each protein mutant to all 64 sequences [44], and plotted this against the corresponding measured 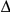 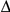 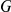 of binding (Figure 6, see Materials and Methods). Theoretically sites with an 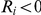 bits are predicted to be bound non-specifically, as 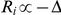 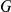 [9], [44].

For all mutants, except for SAR, predicted binding strength is highly correlated with actual binding for sites 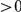 bits (blue sequences in Figure 6), and is poorly correlated for sites 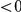 bits (red sequences in Figure 6). The experimental measurement of binding affinity for weakly bound sites has previously been shown to be less accurate than for strongly bound ones [9]. Because of this, we are not surprised by the weak correlation for the sites with an 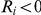 bits. If these sequences are truly bound non-specifically though, we would also expect the slope of the regression line to be 0. For WQR, RQR and RTR we observe a slightly negative slope (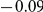, 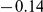 and 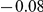 respectively), which suggests that to a small degree, binding energy does change as a function of sequence (bound specifically) for a fraction of these sites. This is evident for RQR, where sites 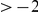 bits lie close to the regression line for the positively bound sequences (Figure 6). We expect the specific/non-specific boundary to be closer to 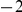 bits for this binding domain. Likewise, for TRR the non-specific boundary is probably at 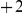 bits, but this deviation from 0 bits can be explained by the low 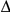 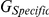, and subsequently biased model for TRR as previously mentioned.

To approximate the non-specific binding energy for each mutant, we determined the intercept of the positive and negative site regression lines (Table 2). SAR appears to be almost completely non-specific from the MITOMI data, and we are not confident in the identified boundary between specific and non-specific binding for this mutant.

Surprisingly, there appears to be a di-nucleotide binding preference for the RTR mutant (Figure 6). RTR binds ‘GC-C’
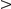
‘GC-T’
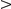
‘GC-G’
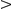
‘GC-A’ and ‘TA-C’
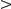
‘TA-T’
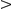
‘TA-G’
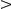
‘TA-A’ with almost equivalent energies between sites that have the same nucleotide at the third position (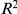 = 0.99). A simple independent and additive model would predict that a single mutation of a ‘G’ to ‘T’ at position 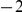 or a ‘C’ to ‘A’ at position 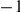 would not affect the binding energy of the site. Indeed, ‘TC-C’
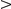
‘TC-T’
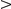
‘TC-G’
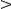
‘TC-A’ and is highly correlated to the equivalent ‘GC-N’ and ‘TA-N’ sites (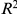 = 0.84 and 0.91 respectively), but ‘GA-N’ sites are not correlated and all sites have a 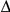 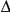 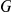 greater than the RTR non-specific binding threshold of 3.30 kJ/mol. This clearly violates a simple independence assumption.

### *In vivo* binding site selection for MarA variants

To identify the *in vivo* binding preferences of the 5 MarA protein variants, we generated a library of selection plasmids for each mutant where positions 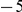, 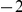, 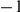, 0 and 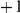 in the *mar* binding site were randomized (Figure 2). We transformed N8453 cells with these libraries and competed them against each other in 5 ml LB+50 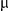
g/ml tetracycline+ 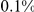 L-arabinose for 24 hours. The competed populations were mini-prepped and sequenced in a single sequencing reaction (Figure S2). Sequence logos were generated for all mutants as described in Materials and Methods (Figure 7). Higher affinity binding sites should be more fit and represented at a higher frequency in the competed population [19]. While the relative peak height for a given base at a given position within the chromatogram is correlated with the base frequency in the population, it can be biased by the identify of the neighboring bases. Therefore, this is a semi-quantitative representation of positional nucleotide frequency.

*In vivo* binding preferences identified by this selection method are consistent with our MITOMI results. The wild type MarA protein (WQR) requires a ‘C’ at position 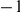 and shows a strong preference for a ‘G’ at position 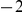. Unlike the MITOMI data, there is more variability at position 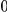 in the selected sites, resulting in a large Kullback-Leibler Divergence between the corresponding WQR logos of 1.67, but a decrease in KLD between WQR and the RQR and RTR mutants (Table S1). The RQR *in vivo* selected sites have an increased variability at positions 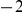 and 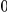 relative to the MITOMI data, but overall the resulting logos are nearly identical (KLD = 0.15). Similar results are observed for RTR (KLD = 0.15), which only shows a slight decrease in degeneracy at position 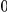 in the experimentally selected sites. Interestingly ‘A’ is not observed at position 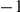 in the RTR *in vivo* sites, even though ‘TAA’ is tightly bound according to the MITOMI data.

The SAR mutant shows substantially less variability in the *in vivo* binding site selection as compared to the MITOMI data; the 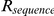 for positions 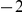 to 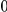 = 4.66 and 0.80 bits respectively. The concentration of tetracycline used for selection, imposes an energetic minimum that the factor must bind its site above to be viable [19]. This lack of variability in the SAR *in vivo* binding site selection suggests that unlike WQR, RQR and RTR, few SAR sites are above this threshold (*i.e.* weakly bound). SAR is the only mutant to show a strong preference for ‘G’ at position 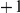, while all other mutants preferred a cytosine there. Differences in the SAR binding preferences observed *in vivo* and *in vitro* may also be accounted for by the presence of a unfavorable ‘C’ at position 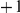 in the MITOMI binding site library (Figure 1), which could significantly reduce the binding affinity of all sites. TRR binds to a single site, ‘GAC’, as expected.

Interestingly, we observed a wide range of degeneracy at position 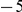, which does not appear to be directly contacted by any of the varied residues. There is a preference for ‘A’ at this position for all mutants, and it is completely conserved for SAR and TRR. We expect that the amount of observed variation at 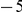 is not dependent upon specific contacts at that base, but on the energetic contribution of the rest of the binding site. That is, weak binding at positions 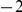, 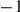 and 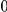 by residual differences requires a base with a higher affinity (‘A’) at position 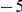 for the site to be sufficiently strong in this selection. This suggests that degeneracy at a single position in a site is not completely defined by the residue that contacts it, but by the energy of the other contacts in the site.

**Figure 8:**
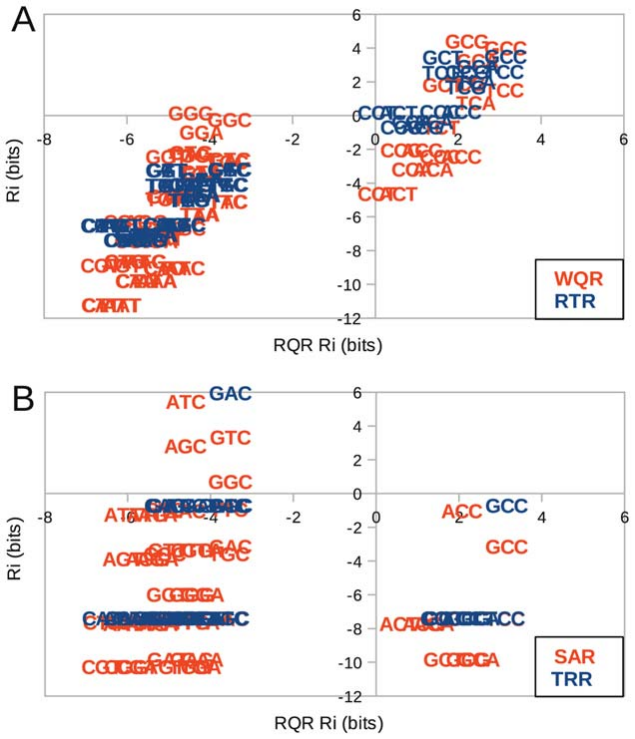
Binding domain mutations can reduce binding targets or generate orthogonal regulators. (A) Comparison of corresponding predicted binding strengths (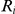) between highly overlapping MarA variants WQR and RTR with RQR. (B) Similar comparison between orthogonal binders SAR and TRR with RQR. The 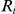 of each mutant to each binding site was calculated using the logos presented in Figure 7 over the range −2 to 0.

To quantify the extent of overlap in sites specifically bound by all mutants *in vivo*, we calculated the predicted binding strength (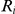) of each mutant to the 64 potential binding site variants at positions 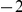 to 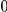, and directly compared these affinities (Figure 8). Since the RQR logo has the lowest information content, we compared all mutants to it. Sequences that fall in the upper right quadrants in Figure 8 are predicted to be specifically bound by the two mutants compared (positive 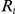 for both). Sites in the lower left are predicted to not be bound by either. The remaining quadrants contain sites that are only bound by one mutant. As the RQR and RTR logos are significantly similar (KLD = 0.14), it is not surprising that their predicted affinities are highly correlated (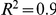). Only a few sequences specifically bound by RQR are not bound by RTR (lower right quadrant) and no unique sequences are bound by RTR (upper left quadrant) suggesting that RTR is merely binding a subset of the sites bound by RQR (Figure 8A). A similar result is observed for WQR, except that it binds a further reduced subset of the specifically bound RQR sites. There is no overlap in specifically bound sites by SAR and TRR with RQR, suggesting that these bind a completely orthogonal set of sequences (Figure 8B).

### Transcriptional output

**Figure 9:**
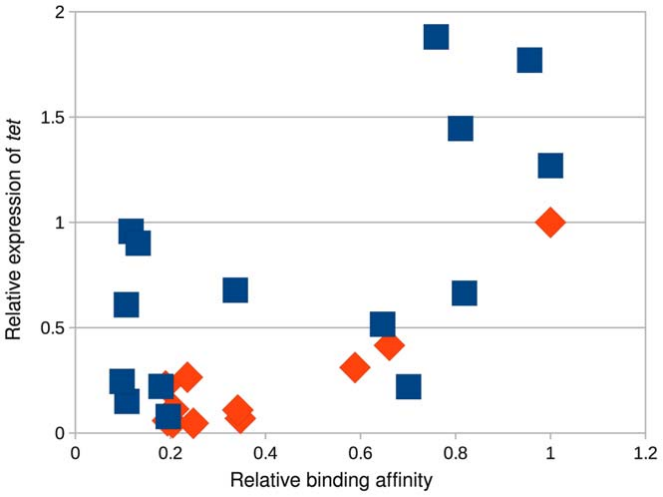
The RQR mutant accesses a much larger output space than wild-type MarA. Relative affinity of a MarA variant for a given site as determined by MITOMI (Figure 4) vs. the quantity of *tet* gene expressed. Expression levels were monitored by Q-PCR. We show data for wild type WQR MarA (orange diamonds) and the RQR mutant (blue squares). The transcriptional output was normalized with wild type MarA bound to its consensus site of ‘GCA’ = 1.

To better understand how mutations in the binding domain affect the transcriptional activity of MarA, we measured the expression of *tet* under the control of wild type MarA (WQR) with 11 different binding sites, and under the control of RQR with 15 different binding sites using quantitative PCR (Figure 9). We chose binding sites for each variant that covered a range of binding strengths based on the MITOMI data. For convenience, we normalized the output so that the relative expression of the ‘GCA’ binding site by WQR is 1.

For the WQR binding sites, the expression data correlate well with binding site strength (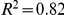 for all sites, 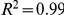 for the 3 tightly bound sites). The non-specifically bound sites show minor variability in their measured output. The expression data for the RQR bound sites do correlate with binding affinity but not as well (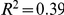 for all sites) and we observed much more variability in the non-specifically bound sites. The transcriptional output from the highest affinity RQR site is almost twice that of the strongest WQR site, suggesting that functionally this mutant can access a much larger dynamic range of outputs.

## Discussion

It is becoming increasingly clear that differences in transcriptional regulation are an important driving force in species diversification and evolution [46], [47]. Fine scale differences in the expression level of an individual gene can be easily achieved by mutations in transcription factor binding sites contained within the associated cis-regulatory region [19]. Larger scale effects on the transcriptional network, and subsequently cellular phenotype, can be accessed through mutations in transcription factor binding domains which will impact the expression levels of all genes within their regulons [48]. As the systematic effects of transcription factor mutations are more difficult to characterize, few experimental studies have been done to probe their evolvability [5]. Since both the informational and regulatory properties of a transcription factor are determined by its binding site energy distribution [1], [20], we developed an *in vivo* selection assay to select for variants with altered binding preferences that still maintain a physiologically relevant transcriptional activity. Further *in vivo* and *in vitro* characterization of a subset of these mutants revealed that a large range of binding preferences, information contents and activities could be accessed with a few mutations suggesting that transcriptional regulatory networks may be easily adaptable.

One way in which regulatory networks are believed to evolve is through the duplication of an existing transcription factor gene that is subsequently selected to recognize a unique set of targets [49], [50]. It is unclear how readily this can happen. Maerkl and Quake observed that a relatively limited range of binding preferences could be accessed by single mutations in the basic helix-loop-helix protein MAX [5]. For MarA, we observed that we could get an orthogonal regulator with two mutations. The double mutant TRR is the most dramatic example. It is absolutely specific for ‘GAC’, which no other variant specifically bound (Figure S1, Figure 8B). Likewise SAR bound its own unique set of sites that do not overlap wild type (Figure 8B). Interestingly, both SAR and TRR have a lower 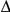 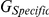 for their highest affinity sites compared to mutants that bind the wild type consensus sequence. This suggests that a novel regulator may emerge or be engineered relatively easily, but may be initially limited in its range of potential activities.

**Figure 10:**
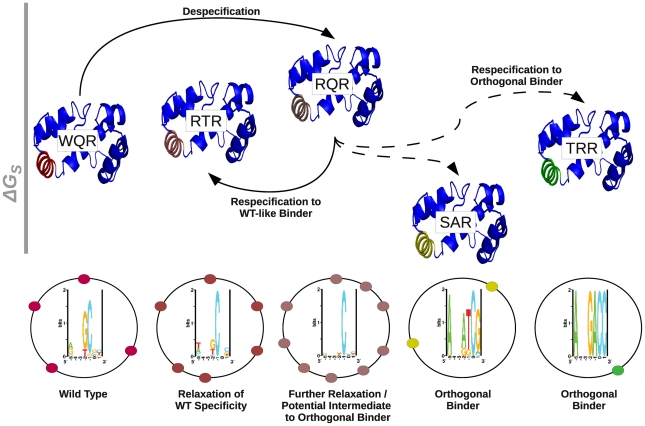
The respecification of an orthogonal regulator may occur through a despecified intermediate. A schematic representation of the respecification of wild type MarA (WQR) to an orthogonal binder through a broadly specific intermediate. Each mutant is represented by the MarA protein structure [28]. Helix 3 in each structure is colored to highlight similarities in binding preferences between mutants. As WQR, RTR and RQR have largely overlapping binding sites, they have a similar coloration. The relative height of each protein structure is determined by the 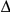 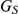 value reported in Table 2. As we are not confident in our estimate for the 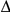 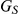 for SAR, we gave it a value of 0 kJ/mol. Solid lines between variants indicate single amino acid differences, dashed lines indicate double mutants. The sequence logo for each variant as determined by the *in vivo* binding site selection assay (Figure 7) are shown directly below that mutant. The black circles surrounding the logo represent the *E. coli* genome, and the colored circles represent hypothetical binding sites for each respective variant.

Gene duplication may not be the only pathway by which orthogonal regulators can evolve. WQR, RQR and RTR appear to have largely overlapping binding sites, where RTR and RQR have an incrementally increasing number of specifically bound sites (Figure 8A). This suggests that a transcription factor could evolve to have an increased or decreased information content (become more or less specific), while still maintaining the majority of its binding targets. An orthogonal regulator could potentially evolve through an intermediate with broader specificity like RQR or RTR (Figure 10). A mutation of this type would impact the relative expression levels of the genes controlled by the transcription factor, as seen in Figure 9, and initially compromise the fitness of the cell [21], but would presumably have a significant advantage over a mutation that leads to the loss of potential targets. Further selection could re-specify the transcription factor after becoming promiscuous to regulate a new set of sequences. As this broadening of specificity can be done relatively easily (WQR can be converted to RQR by a single nucleotide mutation), this pathway may be highly tractable by evolution and useful for engineering regulatory networks. As previously mentioned, the information content of a transcription factor's binding sites is highly correlated to the amount of information needed to specifically locate its binding sites in the genome for bacterial systems [1]. This suggests that as the size of a bacterial factor's regulon increases or decreases, so does the selective pressure on binding site information. The decrease in information from WQR to RTR to RQR, also suggests that a transcription factor can easily evolve to expand or contract the size of its regulon.

The overlap in binding sites between WQR, RQR and RTR may not be surprising as all were selected to bind the wild type consensus sequence ‘GCA’. The dominant feature for these three mutants is a highly conserved ‘C’ at position 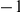 (Figure 5, Figure 7). One possibility is that the 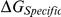 for this base is increased from WQR to RTR to RQR, and a stronger individual contact here compensates for a greater number of energetically unfavorable mismatches at positions 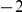 and 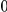, decreasing the information content (Table 2). Interestingly, expanding the number of specifically bound sites for RQR also expands the range of transcriptional outputs nearly two fold (Figure 9). If RQR has a much greater range of potential activities, and largely similar binding preferences to wild type MarA (WQR), why is it not observed in nature? WQR has a greater information content to 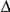 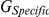 ratio than both RQR and RTR (Table 2), suggesting that it encodes the fewest number of specifically bound sites for its range of binding energies (Table 2). It also appears to have a large energetic gap between its three highest affinity sites and the background, which the other variants lack (Figure 4). These properties of the wild type MarA binding site distribution, and not just overall affinity, may be evolutionarily advantageous and thus selected, as an increased 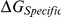 for all sites would decrease the likelihood of the factor binding the wrong location [51], [52], and fewer recognized sites would decrease the probability of spurious sites emerging in the genome [53]. Directly assaying the global effects of these mutations by RNA profiling and chromatin immunoprecipitation would dramatically improve our understanding of their cellular implications.

## Materials and Methods

### MarA selection system and library construction

We modified the plasmid-based selection system described in [19] to select for and characterize MarA variants that have altered binding preferences (Figure 2). Griffith *et al.* generated an L-arabinose inducible MarA expression pBAD18 variant (pBAD18-hisMarA) [34]. We cloned the *marA* gene, the AraC regulated promoter and the *araC* gene from this plasmid into our pBR322-based selection system, allowing for us to control the expression of MarA by the addition of L-arabinose (Figure 2A). An XhoI site was introduced about 10 residues upstream of the start of helix 3 by modifying the ‘CTG’ codon encoding the leucine at residue 30 to the synonymous codon ‘CTC’ by QuickChange [54] (Figure 2). An AgeI site exists immediately downstream of helix 3. To make this a unique restriction site, we removed a second AgeI site present in a non-regulatory region upstream of the *marA* gene by QuickChange.

To generate variants of the MarA-activated *tet* promoter (Figure 2B), the selection plasmid was simultaneously digested with EcoRI and ClaI restriction enzymes for 2 hours at 37

C (NEB). Inserted promoter variants and libraries were generated by DNA synthesis (Integrated DNA Technologies). We synthesized both strands of the DNA, and designed oligos to contain the appropriate overhang to be cloned into the EcoRI and ClaI sites. Digested plasmid and synthesized inserts were ligated overnight at 14

C using T4 DNA ligase (NEB).

To generate binding domain variants (Figure 2C), we used a similar method. Plasmid was digested with XhoI and AgeI simultaneously for 2 hours at 37

C (NEB). The digested plasmid was gel purified and ligated to complementary synthesized inserts that had XhoI and AgeI overhangs. To randomize the residues 42, 45 and 46, we synthesized the oligos with an equal mixture of all four bases at the first two positions of the codon, and an equal mixture of ‘G’ and ‘T’ at the third position of the codon to generate a more equal distribution of amino acids at each position. The ligated promoter and binding domain libraries were transformed into DH10B cells, recovered for 1 hour in LB, and plated on 100 ml LB+30 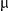
g/ml ampicillin plates. Cells were suspended from the plates in 10 ml LB and mini-prepped using the QIAquick miniprep kit (Qiagen).

### MarA binding domain and binding site selections

To prevent activation of the *tet* gene by the endogenous MarA, Rob or SoxS proteins, selections were performed in the *E. coli* strain N8453 (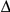 *mar*, 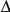 *sox-8*::*cat*, 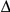 *rob*::*kan* variant of GC4468) prepared by J.L. Rosner and R.G. Martin and obtained from R.E. Wolf. To identify a 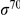 binding site that was only functional when activated, we transformed a library with a variant of the promoter construct shown in Figure 2B that contain the *mar* MarA binding site (Figure 1) and a randomized 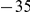 hexamer. These plasmids also contain the wild type MarA protein. The library was transformed in N8453 cells by electroporation, recovered for 1 hour in 500 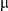
l LB at 37

C, shaken at 225 rpm and plated on 5 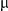
g/ml tetracycline LB plates +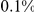 L-arabinose. Individual colonies were picked and streaked on on 10, 15 and 20 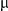
g/ml tetracycline LB plates+/−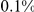 L-arabinose. Colonies that only grew on L-arabinose containing plates were sequenced.

To identify binding domain variants that specifically bound different DNA sequences, libraries were transformed into N8453 cells by electroporation, recovered for 1 hour in 500 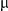
l LB at 37

C, shaken at 225 rpm and plated on 100 ml LB plates containing 30 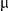
g/ml ampicillin +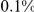 L-arabinose. Colonies that grew on the plates overnight were suspended in 10 ml LB containing 30 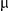
g/ml ampicillin +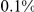 L-arabinose and grown at 37

C, shaken at 225 rpm for 8 hours. 70 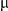
l of these cells were then plated on 25 ml LB agar plates containing 20 or 30 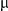
g/ml of tetracycline +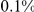 L-arabinose. Individual colonies were picked, grown overnight, miniprepped by the QIAquick miniprep kit and sequenced.

To identify the binding domain for each site that could produce the most *tet* transcript, libraries were transformed by electroporation into N8453 cells and plated on 5 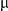
g/ml tetracycline LB plates and grown overnight. These colonies were suspended in 5 ml LB with 5 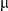
g/ml tetracycline +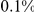 L-arabinose and allowed to grow in liquid culture overnight. The following morning fresh 5 ml 30 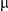
g/ml tetracycline +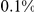 L-arabinose cultures were inoculated with 100 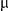
l of the overnight culture and competed for 24 h. The competed library was miniprepped by a QIAquick miniprep kit, transformed into DH10B cells and plated on 30 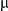
g/ml ampicillin plates. Individual colonies were picked, grown up overnight, miniprepped and sequenced as described above.

Binding site competitions for the 5 MarA selected variants were performed as described previously [19], except that the libraries were transformed into N84533 cells and all media contained 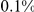 L-arabinose. Libraries were competed in 50 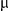
g/ml tetracycline for 24 hours and sequenced on a 96 capillary 3730xl DNA Analyzer (Applied Biosystems). Nucleotide variation in the population of competed promoters was visualized using Finch TV (Geospiza Inc). To generate sequence logos from these data (Figure 7), we measured the peak height of each base at each position in a chromatogram (Figure S2), and divided this height by the summed heights of all peaks at the position to calculate a relative nucleotide frequency. A standard position weight matrix was generate from these frequencies, and represented as a sequence logo using the Delila programs [39].

### MITOMI data acquisition and analysis

MITOMI (Mechanically Induced Trapping of Molecular Interactions) was performed according to Maerkl *et al.* [8]. The 64 variants of the *mar* binding site (Figure 1) were synthesized by Integrated DNA Technologies. *In vitro* transcription and translation was done using the RTS *E. coli* HY kit (Roche). Fluorescently labeled lysines were incorporated into the protein during *in vitro* translation by addition of tRNA-lys-bodipy-fl (Promega). Protein and DNA fluorescence was measured using Genepix (Molecular Devices).

The 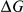 of binding for each variant to each binding site was calculated using 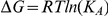, where 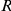 is the ideal gas constant, 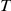 is the temperature of the experiment (295K) and 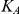 is the association constant as measured by MITOMI. The 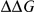 of binding was calculated for each binding site by subtracting the 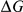 of binding for that site from the 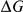 of binding from the highest affinity site for a protein variant.

To generate the energy logos, we calculated a 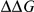 matrix for each variant by determining the difference in binding energy between the strongest bound site for that factor (the consensus site) and all single base-pair mutants. For example, to calculate the relative weights of each base at position 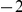 for wild type MarA, we subtracted the measured binding energies of ‘ACA’, ‘CCA’, ‘GCA’ and ‘TCA’ from ‘GCA’. We used the enoLogos webserver to convert these energies into a log-likelihood matrix [42] and generated logos using the Delila programs [39]. The 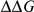 matrices for all logos are given in Table S1.

### Comparison and applications of binding models

To quantify the similarity in binding preferences between MarA variants, we used the program MatCompare to calculate the Kullback-Leiber Divergence (KLD) between the inferred sequence logos [43]. All pair-wise KLD values are reported in Table S1. The relative affinity (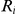) of a given binding model to all DNA sequences was calculated using the information theory based program Scan [44].

### Q–PCR

A library of *mar* binding sites was cloned into plasmids containing either the wild type MarA protein, or the RQR mutant. The library was transformed into N8453 cells, plated on 30 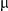
g/ml ampicillin and grown overnight. Individual colonies were grown overnight in 5 ml LB+30 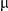
g/ml ampicillin. Glycerol was added to 200 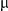
l of cells to a final concentration of 20% and stored at 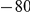
C. The remaining culture was mini-prepped and sequenced to determine which binding site was present. 11 different binding sites covering a range of affinities as determined by MITOMI were chosen for wild type MarA and 15 were chosen for the RQR mutant. These were not the same sites for both factors.

Cultures were inoculated with the frozen samples and grown overnight in 5 ml LB cultures with 30 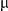
g/ml ampicillin and 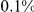 L-arabinose. A fresh 5 ml LB+30 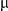
g/ml ampicillin+L-arabinose culture was started at 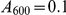 and grown to an 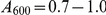. 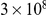 cells were added to RNAprotect Bacteria reagent (Qiagen), and RNA was purified using the RNeasy Mini kit with on-column DNase digestion (Qiagen). cDNA was made from 2 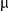
g of RNA using the Superscript III RT kit (Invitrogen). QPCR was performed with the SYBR green mix from NEB. QPCR primers specific to the *tet* and *marA* gene were both used. The relative expression of the *tet* gene was determined by the ratio of *tet* abundance over *marA* abundance for each sample.

## Supporting Information

Figure S1 Bar graph representation of MITOMI data. Bar graph representation of Figure 4.
(EPS)

Figure S2
*In vivo* binding site selection data for 5 MarA variants. Chromatograms show the results from an *in vivo* binding site selection. Five bases (position number in red) were randomized in the MarA binding site (Figure 1), and functional binding sites were selected against each protein variant at 50 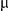
g/ml of tetracycline. All surviving cells were sequenced in a single reaction. The relative height of each peak is a qualitative representation of the frequency of that base at that position in the binding site. The positions of the bases according to the sequence logo (Figure 1) are given at the bottom. Green is ‘A’, Blue is ‘C’, Black is ‘G’ and Red is ‘T’. Positions −4 and −3 were not randomized and therefore are always ‘T’ and ‘G’ respectively.(EPS)

Table S1Supporting data. Sheets 1 and 2: The measured associations (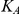) and free energies of binding (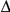 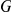) of the five MarA variants to 64 different binding sites. Sheet 3: Binding sites for each mutant are ranked based on their 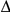 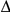 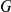 of binding relative to the high affinity site. Sheet 4: The energy matrices used to create Figure 5. Sheet 5: The frequency matrices used to create Figure 7. Sheet 6: Kullback-Leibler Divergences between mutant binding matrices.(XLS)
